# Insulin resistance and β-cell dysfunction in individuals with normal glucose tolerance but elevated 1-h post-load plasma glucose

**DOI:** 10.3389/fendo.2025.1507107

**Published:** 2025-02-03

**Authors:** Qianwen Nie, Xue Jin, Yahui Mu, Youyuan Huang, Aimei Dong

**Affiliations:** ^1^ Department of General Practice, Peking University First Hospital, Beijing, China; ^2^ Department of Endocrinology, Peking University First Hospital, Beijing, China

**Keywords:** prediabetes, 1-h post-load plasma glucose, insulin resistance, β-cell dysfunction, OGTT (oral glucose tolerance test)

## Abstract

**Objective:**

Diabetes and its complications impose a significant burden on public health, necessitating early identification and intervention, yet current prediabetes diagnostic criteria may not fully capture all high-risk individuals. Evaluate and compare insulin resistance (IR) and β-cell dysfunction in individuals with normal glucose tolerance (NGT) and 1-hour post-load plasma glucose (1-h PG) ≥ 8.6 mmol/L versus < 8.6 mmol/L, as well as prediabetes based on IFG and/or IGT.

**Research design and methods:**

This retrospective study included individuals at risk for diabetes who underwent an Oral Glucose Tolerance Test (OGTT), classified as having NGT or prediabetes according to ADA criteria. IR and β-cell dysfunction were assessed using the Matsuda index, insulinogenic index (IGI30), and oral disposition index (DI).

**Results:**

Among the 9,452 participants, 37.8% had NGT, and 62.2% were IFG or IGT in OGTT. Of the NGT group, 39.2% had a 1-h PG ≥ 8.6 mmol/L, with a higher mean age (53 vs. 47 years for those with 1-h PG < 8.6 mmol/L). Glucose and insulin curves showed that the NGT group with 1-h PG ≥ 8.6 mmol/L exhibited glucose profiles similar to those with isolated impaired fasting glucose (I-IFG), marked by elevated glucose, early insulin secretion impairment, and delayed insulin peaks. Older individuals (≥ 65 years) had higher glucose and a higher prevalence of abnormal 1-h PG but showed no significant differences in IR or β-cell dysfunction compared to younger individuals.

**Conclusions:**

A 1-h PG ≥ 8.6 mmol/L in individuals with NGT is associated with substantial β-cell dysfunction, highlight the value of incorporating 1-h PG measurement into diagnostic assessments for early detection of insulin secretion impairments across age groups.

## Introduction

The International Diabetes Federation (IDF) estimates that in 2021, 537 million adults worldwide were living with diabetes, a number projected to rise to 783 million by 2045 (1). Diabetes and its complications pose a significant burden on public health systems. Early identification and intervention are potential strategies to reduce the incidence of diabetes. However, the definitions and screening criteria for prediabetes differ between guidelines published by different organizations, resulting in estimations of prevalence that can vary widely from one another (2, 3). Understanding prediabetes for early identification and intervention is crucial to potentially reducing progression to diabetes.

Current fasting plasma glucose (FPG) and 2-h plasma glucose (PG) standards for diagnosing prediabetes may not adequately capture all individuals at high risk (4). Based on current criteria, longitudinal studies have shown that 50-60% of individuals with prediabetes do not progress to diabetes within about 10 years, while 30-40% of diabetes patients were actually of normal glucose tolerance (NGT) at baseline (5). In addition, identifying potential diabetic patients among the NGT population is crucial for the prevention, treatment, and diagnosis of diabetes. Prospective cohort studies affirm the role of 1-h PG ≥ 8.6 mmol/L (155 mg/dL) in the early identification of individuals at high risk for type 2 diabetes and its associated complications. Studies indicate that 1-h PG correlates differently with the risk of type 2 diabetes across different ages, genders, and ethnicities among the elderly, suggesting the need for specific risk assessments for these subgroups (6).

Studies show that elevated 1-h PG detects early declines in β-cell function and insulin sensitivity, but which factor is more prominent remains unclear. The RISC study found that NGT with high 1-h PG had a significant decline in insulin sensitivity and β-cell glucose sensitivity (7). And some studies have also shown that the risk of developing type 2 diabetes is significantly increased in the NGT with elevated 1-h PG, suggesting that impaired β-cell function plays an important role in this process (8, 9). An elevation in 1-h PG may reflect specific pathophysiological mechanisms related to IR and β-cell dysfunction.

The present study aimed to investigate the IR and β-cell dysfunction between NGT and subtypes of prediabetes and clarify the variations based on the PG during the oral glucose tolerance test (OGTT) in older individuals within each subgroup of prediabetes, using a large-scale database of Chinese patients with prediabetes.

## Method

### Study population

**Figure 1:**
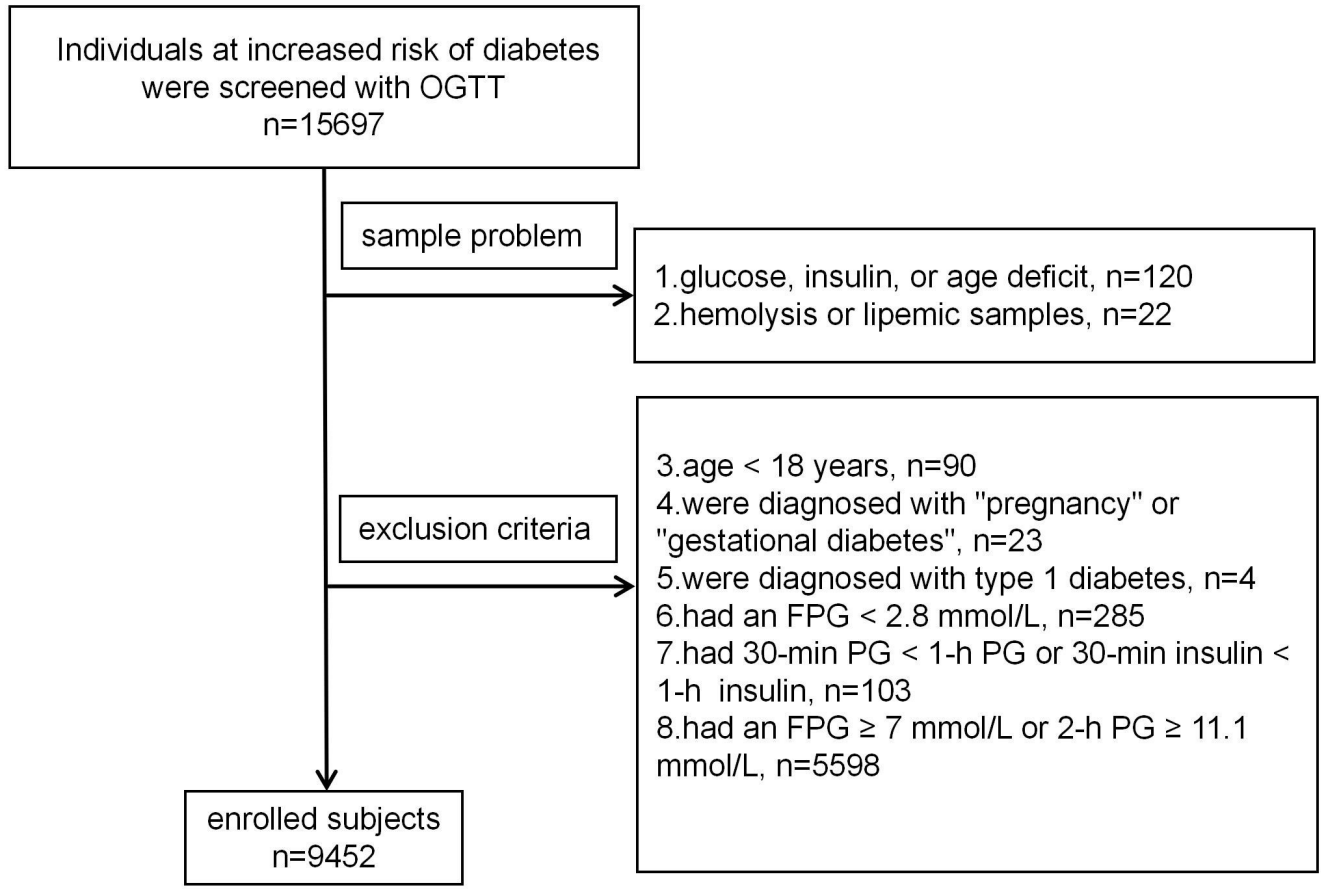
Participants screening flowchart.

A total of 15,697 people, who were mainly from the Han Chinese population and identified by clinicians as at risk for diabetes were screened with a 75-g OGTT between July 1, 2010, and June 30, 2021 (**Figure 1**). People were excluded if they: had missing data on PG, insulin, or age (n=120), had potential hemolysis or lipemic samples (n=22), were under the age of 18 years (n=90), were diagnosed with “pregnancy” or “gestational diabetes” (n=23), were diagnosed with type 1 diabetes (n=4), had an FPG < 2.8 mmol/L (n=285), had 30-minute PG or insulin levels lower than fasting (n=103), or had an FPG ≥ 7 mmol/L or 2-h PG ≥ 11.1 mmol/L (n=5,598). Ultimately, 9,452 participants were included in the analysis.

### The OGTT

After an 8 to 10-hour overnight fast, participants underwent a 75-g OGTT. PG and insulin concentrations were measured at 0, 30, 60 and 120 minutes. No medication was taken on the morning of the test. PG concentration was determined using the glucose oxidase method, while insulin concentration was measured using an electrochemiluminescence immunoassay on the Roche Cobas e601 automatic analyzer. All laboratory analyses were conducted at Peking University First Hospital, Beijing, China. The intra-assay coefficient of variation for insulin measurements was 2.2%, and the inter-assay coefficient of variation was 2.7%.

### Definition of prediabetes

Following the general cut points of the American Diabetes Association (ADA), participants were classified into four groups: (1) NGT, defined as FPG < 5.6 mmol/L and 2-h PG < 7.8 mmol/L; (2) isolated IFG (I-IFG) with FPG in the range 5.6–6.9 mmol/L and 2-h PG < 7.8 mmol/L; (3) isolated IGT (I-IGT) with FPG < 5.6 mmol/L and 2-h PG in the range of 7.8-11.0 mmol/L; (4) Combined IFG and IGT (IFG + IGT).

### IR and β-cell dysfunction

Matsuda index (calculated as 10,000/[(G0×I0×Gmean×Imean)^1/2^]) of insulin sensitivity and the insulinogenic index (IGI30 =ΔI30/ΔG30, disposition index (DI) = IGI30×Matsuda index) were calculated from OGTT and compared with a reference group. The reference group comprised 546 individuals recruited from those who completed the 75-g OGTT during outpatient diabetes screening, with a median age of 39 years (IQR: 32, 53), comprising 175 males and 371 females. Their normal glucose tolerance was defined by the following criteria: FPG < 5.6 mmol/L, 1-h PG < 8.6 mmol/L, 2-h PG < 7.8 mmol/L, and HbA1c < 5.7%. The following exclusion criteria were adopted: severe insulin resistance (fasting insulin > 50 μIU/mL), low fasting insulin (fasting insulin < 3 μIU/mL), delayed insulin peak time (with the insulin peak occurring at 2 hours during the OGTT insulin release test). The FPG, 30-minute PG, 1-h PG, and 2-h PG measured during the OGTT were 4.94 mmol/L (IQR: 4.72, 5.22), 7.84 mmol/L (IQR: 7.24, 8.47), 6.79 mmol/L (IQR: 5.77, 7.62), and 5.66 mmol/L (IQR: 5.02, 6.24) respectively. The insulin levels were recorded as 8.35 μIU/mL (IQR: 6.05, 11.84), 73.43 μIU/mL (IQR: 50.73, 105.73), 67.41 μIU/mL (IQR:48.72, 99.35) and 45.28 μIU/mL (IQR:30.37, 66.03) for FPG, 30-minute PG, 1-h PG, and 2-h PG respectively. If the Matsuda index was less than the lowest 5% of the range of insulin sensitivity in the reference group, the participant was considered to have severe IR; those whose Matsuda index was in the 6-25th percentile had moderate IR; and those whose Matsuda index was above the 25th percentile were insulin sensitive. Severe impairment of insulin secretion was defined as less than 50% of the insulin secretion index, moderate impairment was 50-70%, and normal secretion was more than 70%. Among the normoglycemic subjects, the 5th and 25th percentiles of the Matsuda index were 1.78 and 3.20, respectively. The median values of the 50th and 70th percentiles for the DI were 48.50 and 67.89.

### Data collection and analysis

Data were collected using a standardized form and entered into Excel 2021. Statistical analyses were performed using SPSS version 26, and graphical representations were generated with GraphPad Prism 8.0. Continuous data were assessed for normality using the Shapiro-Wilk test and the Kolmogorov-Smirnov test. Normally distributed data were described using mean ± standard deviation, while non-normally distributed data were described using median and interquartile range (first and third quartiles). For inferential statistics, normally distributed continuous data were analyzed using analysis of variance (ANOVA), and non-normally distributed data were evaluated using the Kruskal-Wallis test, a non-parametric method for comparing independent samples. *Post-hoc* pairwise comparisons were conducted using Bonferroni correction to adjust for multiple comparisons and assess statistical significance. Categorical data were analyzed using the chi-square test. All statistical tests were two-tailed, with a significance level set at α= 0.05.

This study adhered to ethical research standards, excluding irrelevant information and anonymizing data. Ethical approval was granted by the Ethics Committee of Peking University First Hospital (Approval No. 2022-Yan-172-002).

## Results

### Clinical characteristics

A total of 9,452 participants were enrolled in this analysis, with a median age of 54 years (IQR: 43, 61), comprising 3,630 males and 5,822 females. The FPG, 30-minute PG, 1-h PG, and 2-h PG measured during the OGTT were 5.57 mmol/L (IQR: 5.14, 6.04), 9.6 mmol/L (IQR: 8.5, 10.68), 10.15 mmol/L (IQR: 8.3, 11.89), and 7.41 mmol/L (IQR: 6.19, 8.89) respectively. The insulin levels were recorded as 9.79 μIU/mL (IQR: 6.74, 14.4), 56.7 μIU/mL (IQR: 37.56, 85.89), 80.98 μIU/mL (IQR:54.21, 122.8) and 70.39 μIU/mL (IQR:43.34, 112.6) for FPG, 30-minute PG, 1-h PG, and 2-h PG respectively.

### 1-h PG distribution in individuals with NGT and prediabetes

**Figure 2:**
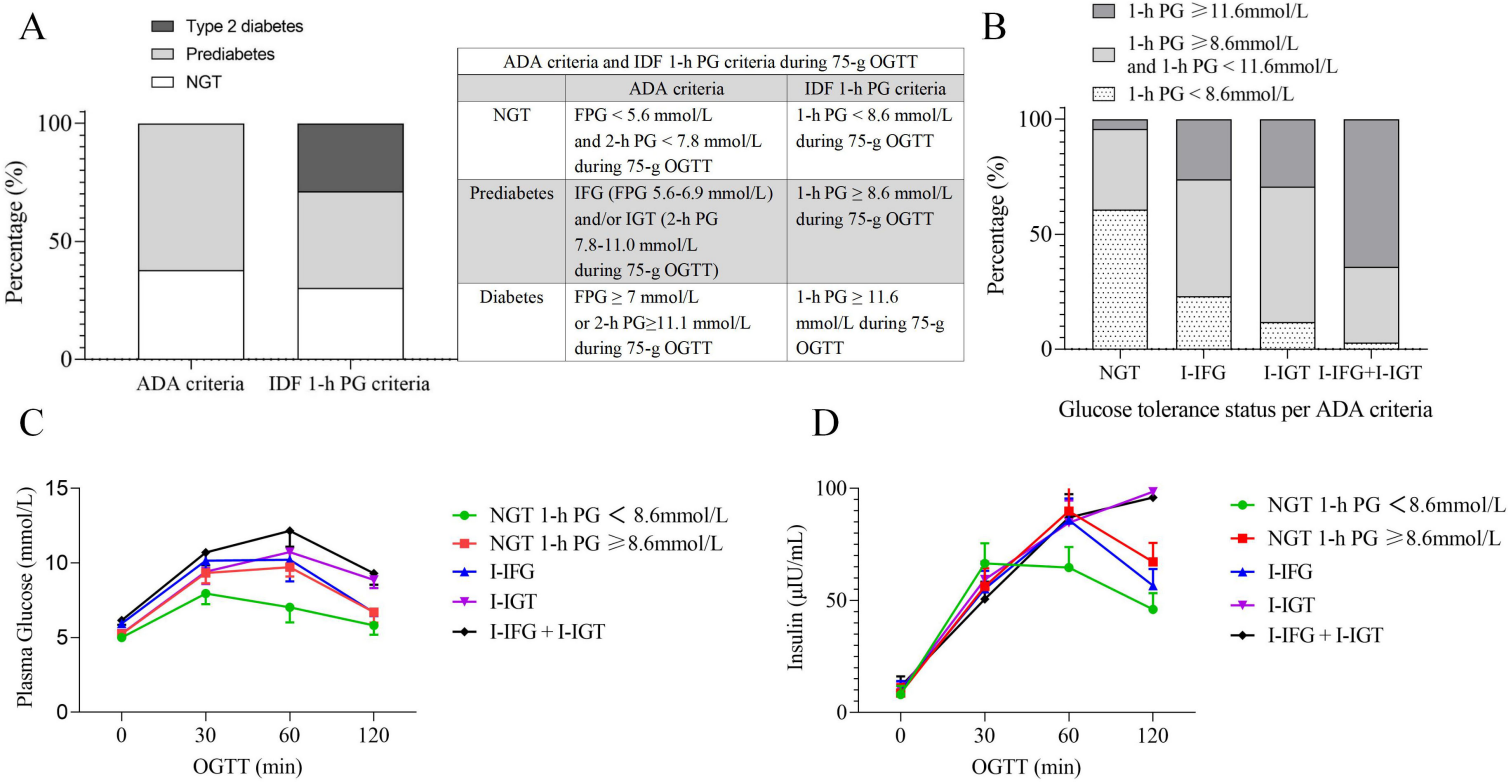
Glucose tolerance status and plasma glucose-insulin profiles based on ADA and IDF 1-h PG Criteria. **(A)** Distribution of glucose tolerance status according to ADA and IDF 1-h PG criteria. **(B)** 1-h PG distribution by IDF 1-h criteria across ADA-defined glucose tolerance statuses. **(C)** The plasma glucose curves during the 75-g OGTT for different glucose tolerance statuses. **(D)** The Insulin curves during the 75-g OGTT for different glucose tolerance statuses. ADA, American Diabetes Association; IDF, The International Diabetes Federation; OGTT, oral glucose tolerance test; NGT, normal glucose tolerance; PG, plasma glucose; I-IFG, isolated impaired fasting glucose; I-IGT, isolated impaired glucose tolerance; I-IFG + I-IGT, combined I-IFG and I-IGT; min, minutes.

According to the IDF 1-h PG diagnostic criteria, there were 2,855 individuals (30.2%) classified as having NGT, 3,886 individuals (41.1%) classified as having prediabetes, and 2,711 individuals (28.7%) classified as having type 2 diabetes (**Figure 2A**). According to the ADA diagnostic criteria, 3,577 individuals (37.8%) were classified as having NGT, while 5,857 individuals (62.2%) were classified as having prediabetes. The prediabetes includes 1,933 individuals (20.5%) in the I-IFG group, 1,372 individuals (14.5%) in the I-IGT group, and 2,570 individuals (27.2%) in the combined I-IFG + I-IGT group (**Figure 2B**). These data indicate substantial differences in prevalence rates for NGT, prediabetes, and type 2 diabetes depending on the diagnostic criteria used (ADA vs. IDF 1-h PG).

### IR and β-cell dysfunctional status in different glucose metabolism status

Among the 3,577 individuals identified with NGT (comprising 1,137 men and 2,440 women), 39.2% of the NGT population, had a 1-h PG ≥ 8.6 mmol/L (**Table 1**). The NGT group with a 1-h PG ≥ 8.6 mmol/L demonstrates an older age profile compared to those with a 1-h PG < 8.6 mmol/L (median ages: 53 vs. 47), which approaches the age distribution typically observed in prediabetic groups.

**Table 1:** The clinical characteristics and results of the OGTT in participants with different glucose metabolism status.

	NGT 1-h PG <8.6 mmol/L n = 2174	NGT 1-h PG ≥8.6 mmol/L n = 1403	I-IFG n = 1933	I-IGT n = 1372	I-IFG+I-IGT n = 2570
Age, (years)	47 (34, 58)	53 (41, 61) ^* †^	55 (47, 61) ^*†^	54 (42, 63) ^*†^	56 (47, 63) ^*†^
Female (%)	1612 (74.1)	828 (59.0) ^*†^	1064 (55.0) ^*†^	898 (65.5) ^*†^	1420 (55.3) ^*†^
IGI30	21.45 (13.33, 33)	11.79 (7.77, 18.37) ^*^	10.93 (6.72, 17.52) ^*†^	12.04 (7.39, 18.41) ^*^	8.59 (5.37, 14.03) ^*†^
Matsuda index	4.64 (3.19, 6.39)	3.61 (2.44, 5.14) ^*^	3.13 (2.21, 4.39) ^*†^	3.11 (2.08, 4.61) ^*†^	2.52 (1.78, 3.61) ^*†^
DI	91.4 (67.19, 134.12)	42.91 (32.08, 55.7) ^*^	33.06 (22.31, 49.36) ^*†^	36.7 (25.56, 50.3) ^*†^	21.66 (15.75, 30.28) ^*†^

Data following a non-normally distributed data are described using the median (interquartile range). NGT, normal glucose tolerance; PG, plasma glucose; I-IFG, isolated impaired fasting glucose; I-IGT, isolated impaired glucose tolerance; n, sample size; IGI30, early insulin secretory index; Matsuda Index, composite whole-body insulin sensitivity index; DI, disposition index. ^*^P < 0.05 compared with NGT 1-h PG < 8.6 mmol/L. ^†^P< 0.05 compared with NGT 1-h PG ≥ 8.6 mmol/L.

The OGTT PG curves indicate that the NGT group with a 1-h PG ≥ 8.6 mmol/L exhibits a PG profile similar to that of the I-IFG group. Their PG at each point were higher than those in the NGT group with a 1-h PG < 8.6 mmol/L but slightly lower than in the I-IGT group (**Figure 2C**). The insulin curves during the OGTT revealed that the insulin profile in the NGT group with a 1-h PG ≥ 8.6 mmol/L is similar to that observed in the I-IFG group. Insulin secretion at 30 minutes was lower than in the NGT group with a 1-h PG < 8.6 mmol/L, while insulin secretion at 1 h and 2 h was modestly higher than in the I-IFG group (**Figure 2D**). These findings suggest that the NGT group with a 1-h PG ≥ 8.6 mmol/L exhibits a degree of IR and β-cell functional impairment.

Regarding the insulinogenic index (IGI30), there was no significant statistical difference between the NGT group with a 1-h PG ≥ 8.6 mmol/L and the I-IGT group (values: 11.79 [7.77, 18.37] vs. 12.04 [7.39, 18.41], p = 0.138). However, their IGI30 was higher than those of both the I-IFG (values: 11.79 [7.77, 18.37] vs. 10.93 [6.72, 17.52], p < 0.001) and I-IFG+I-IGT groups (values: 11.79 [7.77, 18.37] vs. 8.59 [5.37, 14.03], p < 0.001), but significantly lower than the NGT group with a 1-h PG < 8.6 mmol/L (values: 11.79 [7.77, 18.37] vs. 21.45 [13.33, 33], p < 0.001). Specifically, the IGI values were approximately 45.03% lower in the NGT group with a 1-h PG ≥ 8.6 mmol/L, with further decreases observed in the I-IFG and I-IFG+I-IGT groups by 49.04% and 59.95%, respectively.

The Matsuda index, which indicates insulin sensitivity, was lower in the NGT group with a 1-h PG ≥ 8.6 mmol/L compared to those with a 1-h PG < 8.6 mmol/L (values: 3.61 [2.44, 5.14] vs. 4.64 [3.19, 6.39], p < 0.001), yet it was higher than that observed in the I-IFG (values: 3.61 [2.44, 5.14] vs. 3.13 [2.21, 4.39], p < 0.001), I-IGT (values: 3.61 [2.44, 5.14] vs. 3.11 [2.08, 4.61], p < 0.001), and I-IFG+I-IGT groups(values: 3.61 [2.44, 5.14] vs. 2.52 [1.78, 3.61], p < 0.001). The Matsuda index in the NGT group with a 1-h PG ≥ 8.6 mmol/L did not fall below 3.20, suggesting retained insulin sensitivity, whereas the I-IFG, I-IGT, and I-IFG+I-IGT groups exhibited moderate IR.

The oral DI, indicative of insulin secretion capacity, showed severe impairment across the NGT group with a 1-h PG ≥ 8.6 mmol/L (values: 42.91 [32.08, 55.7]), the I-IFG (values: 33.06 [22.31, 49.36]), I-IGT (values: 36.7 [25.56, 50.3]) and I-IFG+I-IGT groups (values: 21.66 [15.75, 30.28]), all exhibiting values below 48.50. These values represent significant reductions compared to the NGT group with a 1-h PG < 8.6 mmol/L (values: 91.4 [67.19, 134.12], p < 0.001), with decreases of 53.05%, 63.83%, 59.85%, and 76.30%, respectively, highlighting a more pronounced impairment in insulin secretion capability.

### Assessment of OGTT PG abnormality thresholds at different time points for detecting severe β-cell dysfunction (DI < 48.5)

For detecting severe impairment of insulin secretion (DI < 48.50), compared with FPG ≥ 5.6 mmol/L and 2-h PG ≥ 7.8 mmol/L, 1-h PG ≥ 8.6 mmol/L demonstrated significantly higher sensitivity, at 94.60% compared to 65.60% and 58.00%, respectively. While the 1-h PG had lower specificity (69.50%) compared to FPG (80.90%), it was higher than the specificity observed at the 2-h mark (69.50%). The positive likelihood ratio (LR+) for 1-h PG was 3.102, which is higher than that of 2-h PG (1.902) but slightly lower than that of FPG (3.434). The negative likelihood ratio (LR-) for 1-h PG was markedly better at 0.078 compared to 0.425 for FPG and 0.604 for 2-h PG. Furthermore, the diagnostic odds ratio (DOR) for 1-h PG was exceptionally higher at 39.919, compared to 8.077 for FPG and 3.147 for 2-h PG, indicating a substantially better diagnostic performance. The accuracy of 1-h PG (84.88%) was also superior to that of 2-h PG (68.11%), though only slightly higher than FPG (71.55%). These results suggest that the 1-h PG measurement could provide a more sensitive and effective means for diagnosing β-cell dysfunction, albeit with some compromise in specificity compared to FPG.

### IR and β-cell dysfunction across different age groups in individuals with NGT

Nearly 50% of the NGT population aged 65 years and older exhibited a 1-h PG ≥ 8.6 mmol/L. In contrast, 37.8% of patients under 65 years of age demonstrated a 1-h PG ≥ 8.6 mmol/L. In individuals with NGT, differences in glycemic responses and indices of insulin sensitivity and secretion were examined across different age groups. For those with a 1-h PG < 8.6 mmol/L, there were no statistically significant differences in the IGI30, Matsuda Index, or DI between the age groups. However, in individuals aged 65 and above, PG was higher compared to those under 65 years. Among individuals with a 1-h PG ≥ 8.6 mmol/L, the only age-related difference that reached statistical significance was in the Matsuda Index. Specifically, this index was slightly higher in individuals aged 65 years and older (**Table 2**).

**Table 2:** Insulin resistance and β-cell dysfunction across different age groups in individuals with NGT.

	<65 years		≥65 years	
	NGT 1-h PG < 8.6 mmol/L	NGT 1-h PG ≥ 8.6 mmol/L	NGT 1-h < 8.6 mmol/L	NGT 1-h PG ≥ 8.6 mmol/L
n(%)	1949 (62.19)	1185(37.81)	225(50.79)	218(49.21)
male(%)	482(24.7)	497(41.9)	80(35.6)	78(35.8)
PG 0-h (mmol/L)	5.00(4.75,5.25)	5.24(4.98,5.41) ^*^	5.14(4.91,5.33) ^*^	5.26(5,5.46) ^#^
PG 0.5-h (mmol/L)	7.93(7.22,8.61)	9.33(8.66,10) ^*^	8.15(7.45,8.72) ^*^	9.35(8.53,10.22) ^#^
PG 1-h (mmol/L)	7.02(5.98,7.81)	9.72(9.11,10.64) ^*^	7.28(6.21,7.92) ^*^	9.68(9.1,10.87) ^#^
PG 2-h (mmol/L)	5.81(5.18,6.51)	6.68(6.01,7.24) ^*^	6.07(5.32,6.82) ^*^	6.9(5.98,7.37) ^#^
INS 0-h (μIU/ml)	8.13(5.83,11.94)	8.96(6.25,13.57) ^*^	7.45(5.15,10.67) ^*^	7.89(5.35,10.47) ^‡^
INS 0.5-h (μIU/ml)	66.68(45.37,100.1)	58.24(37.45,87.57) ^*^	64.72(44.58,95.96)	52.64(37.26,78.91) ^#^
INS 1-h (μIU/ml)	64.7(45.39,94.49)	91.44(60.17,140) ^*^	64.78(43.32,94.63)	81.54(61.98,121.9) ^#^
INS 2-h (μIU/ml)	45.87(30.55,69.18)	68.03(42.47,97.93) ^*^	49.14(29.26,76.75)	57.74(42.12,90.54) ^#^
IGI30	21.49(13.6,33.3)	11.93(7.77,19) ^*^	20.61(12.93,30.67)	11.24(7.62,15.98) ^#^
Matsuda index	4.63(3.19,6.39)	3.54(2.34,5.11) ^*^	4.67(3.23,6.52)	3.96(2.74,5.63) ^#‡^
DI	91.83(67.01,134.65)	42.8(31.76,55.22) ^*^	89.38(69.42,126.37)	43.69(33.6,57.46) ^#^

NGT, normal glucose tolerance; PG, plasma glucose; INS, insulin; IGI30, early insulin secretory index; Matsuda Index, composite whole-body insulin sensitivity index; DI, disposition index. ^*^P < 0.05 compared with NGT 1-h PG < 8.6 mmol/L in individuals under 65 years old, ^#^P < 0.05 compared with NGT 1-h PG < 8.6 mmol/L in individuals aged 65 years and above, ^‡^P < 0.05 compared with NGT 1-h PG ≥ 8.6 mmol/L in individuals under 65 years old.

Among those under 65, when comparing PG ≥ 8.6 mmol/L to those < 8.6 mmol/L, there was a reduction of approximately 44.58% in IGI30, 23.54% in Matsuda Index, and 53.39% in DI. In individuals aged 65 and above, similar declines were observed with reductions of approximately 45.45% in IGI30, 15.20% in Matsuda Index, and 51.11% in DI. Despite reductions in both insulin secretion and sensitivity in higher PG contexts, the extent of decline varied between the two age groups. Notably, the Matsuda Index, which measures insulin sensitivity, showed a lesser decline in the older age group (approximately 15.20%) compared to the younger group (approximately 23.54%). This may indicate a more pronounced decrease in insulin sensitivity among the younger individuals compared to their older counterparts. Among individuals aged 65 and above belonging to the NGT group with a 1-h PG ≥ 8.6 mmol/L, there were no statistically significant differences in the early insulin secretion index or the oral DI. Although the Matsuda index was higher in the ≥ 65 years group compared to those under 65, it did not fall below 3.20 in any group (**Table 2**).

The 1-h PG ≥ 8.6 mmol/L appears to be more accurate in diagnosing severe impairment of insulin secretion. Incorporating 1-h PG into diagnostic assessments can facilitate early identification and management of β-cell dysfunction across diverse populations, regardless of age.

## Discussion

In this study, the proportion of participants within the NGT group exhibiting a 1-h PG ≥ 8.6 mmol/L was approximately 39.22%. Comparative data from other studies indicate varying prevalence rates of 1-h PG abnormalities within NGT populations: 16.7% in the San Antonio Heart Study (10), 15.8% in the Botnia Study (11), and 39.0% in the GENFIEV study (12). These discrepancies largely reflect differences in study populations. The San Antonio Heart Study and the Botnia Study were based on general populations, whereas the GENFIEV study included individuals at risk for diabetes, such as those with a family history of diabetes or dyslipidemia. Our study primarily included individuals suspected of diabetes with mild glucose abnormalities or high-risk factors for diabetes, who underwent an OGTT following their own inclination and upon the recommendation of healthcare professionals. Hence, the prevalence of 1-h PG abnormalities in our study was similar to that in the GENFIEV study, reflecting a higher prevalence among at-risk individuals with NGT. This underscores the importance of considering 1-h PG measurements in the early detection of potential diabetic conditions in high-risk groups.

The RISC study found that there was a progressive and significant decline in insulin sensitivity and β-cell glucose sensitivity (i.e., representing the dependence of insulin secretion on absolute glucose concentration at any time point during the OGTT) as one progresses from NGT with normal 1-h PG to NGT with high 1-h PG, and to individuals with IGT while basal and total insulin secretion significantly increased (7). Our research revealed that compared to those with NGT and a 1-h PG < 8.6 mmol/L, participants with NGT and a 1-h PG ≥ 8.6 mmol/L exhibited a 45.03% reduction in early insulin secretion index (IGI30), a 22.20% decrease in insulin sensitivity (Matsuda index), and a 53.05% decline in β-cell function index (DI). Marini et al. conducted a study on Caucasians with elevated 1-h PG using a high insulin-euglycemic clamp (HEC) to assess IR (glucose disposal rate, M value, mg·kg^−1·min^−1), considered the gold standard for evaluating insulin sensitivity (13). Their research also utilized an intravenous glucose tolerance test (IVGTT) to measure acute insulin response (AIR), followed by calculation of the DI (calculated as MFFM * AIR) to evaluate β-cell function. The findings indicated that compared to those with NGT and a 1-h PG < 8.6 mmol/L, individuals with NGT and a 1-h PG ≥ 8.6 mmol/L experienced a 20% reduction in insulin sensitivity and a 58% decline in β-cell function. Our study utilized an OGTT to calculate the Matsuda index for assessing insulin sensitivity and the DI to evaluate β-cell function. Both studies yielded similar conclusions, indicating that participants with NGT but elevated 1-h PG already exhibit a decrease in insulin sensitivity and approximately a 50% reduction in β-cell function.

Type 2 diabetes exhibits heterogeneity in both clinical manifestations and progression, with this heterogeneity being shown as the variations among individuals in blood glucose metabolism processes and the risk of complications (14). There are differences in insulin resistance and β-cell dysfunction between the NGT with 1-h PG ≥ 8.6 mmol/L and those with IFG and/or IGT. It has been reported that both individuals with NGT having a 1-h PG ≥ 8.6 mmol/L and those with IGT exhibited a comparable impairment in insulin sensitivity, and there was no significant disparity in the DI between them. Nevertheless, only the NGT with 1-h PG ≥ 8.6 mmol/L manifested a deficiency in the first-phase insulin secretion as evaluated by the IVGTT (13). Other studies indicate that compared to the NGT with a 1-h PG ≥ 8.95 mmol/L group, the IGT group exhibits lower insulin sensitivity and higher insulin secretion (7). Our research reveals that NGT with 1-h PG ≥ 8.6 mmol/L group already exhibit a decline in early insulin secretion that is equivalent to that of individuals in I-IGT group. However, early insulin secretion (IGI30) tends to decrease when compared to the I-IFG and I-IFG+ I-IGT groups. Additionally, in the NGT with a 1-h PG ≥ 8.6 mmol/L group, insulin sensitivity (Matsuda index) and β-cell function (DI) show a declining trend when compared to the I-IFG group, I-IGT group, and I-IFG+ I-IGT group.

In a study of obese adolescents (15), it was found that even among adolescents classified as NGT but with higher 2-h glucose levels, β-cell function was impaired relative to insulin sensitivity. This indicates that in the obese adolescent population, an imbalance between β-cell function and insulin resistance already exists in the pre-diabetic stage, and β-cell dysfunction may precede insulin resistance. Among Japanese patients with IFG, it was determined that both the reduction in early insulin secretion and the decline in insulin sensitivity contribute to the deterioration of blood glucose levels, with impaired early insulin secretion playing a more crucial role in the elevation of postprandial blood glucose (16). The findings of our research on the Chinese population indicate that individuals with NGT and a 1-h PG ≥ 8.6 mmol/L exhibit significant β-cell dysfunction and it is more prominent than insulin resistance. Our finding shows certain similarities and associations with previous research results in other populations. This further attests to the significance of β-cell dysfunction in the pre-diabetic stage across different populations and also reflects the intricate relationship between β-cell dysfunction and insulin resistance during the progression of the disease.

Our analysis supports the growing body of evidence emphasizing the diagnostic efficacy of the 1-h PG during an OGTT for detecting both diabetes and prediabetes. The use of FPG, 1-h PG, and 2-h PG as diagnostic markers has been extensively validated, with 1-h PG thresholds demonstrating significant diagnostic accuracy due to their high sensitivity and specificity, particularly at the 8.6 mmol/L threshold. Jagannathan et al. provided a comprehensive historical perspective on the OGTT, reiterating its relevance a century after its introduction and underscoring the pivotal role of 1-h PG in enhancing diagnostic precision (17). This is corroborated by Buysschaert et al., who identified the 1-h PG as a critical indicator for the early detection of prediabetic states, highlighting its potential to facilitate earlier interventions (18). Furthermore, Bergman et al. reviewed various methodologies for detecting glycemic disorders, noting that 1-h PG is a valuable component of a multi-parametric approach in diabetes screening and diagnosis (19). Our findings also suggest that the use of 1-h PG for screening prediabetes is particularly effective in younger individuals, given the high sensitivity and notable specificity of the 8.6 mmol/L threshold in this age group.

The reason for supporting the use of 1-h PG as a diagnostic marker is the early detection of β-cell dysfunction. Some researches highlight that individuals with abnormal 1-h PG, even within the NGT range, show a 40%-50% reduction in β-cell function compared to healthy individuals, underscoring 1-h PG’s potential in identifying early glycemic impairment (20, 21). Our research has found that the β-cell function in the group with NGT 1-h PG ≥ 8.6 mmol/L is reduced by approximately 50% compared to that in the group with NGT 1-h PG < 8.6 mmol/L. Additionally, our findings suggest that specific 1-h PG thresholds could be superior in the early detection of diabetes, offering a valuable tool in clinical diagnostics with robust accuracy and likelihood ratios. Among high-risk individuals, the 1-h PG ≥ 11.6 mmol/L during OGTT has demonstrated good sensitivity and specificity for detecting type 2 diabetes (22). Our data showed that with diagnostic criteria including the 1-h PG ≥ 11.6 mmol/L during OGTT, more individuals (about 15.8%) would be diagnosed with type 2 diabetes. These results warrant further exploration and validation in diverse patient populations.

The limitation of this study lies in the selection of the reference group for establishing the norms of the Matsuda Index and DI. The subjects, who were sourced from outpatient clinics and identified as being at risk for diabetes, may not constitute a truly representative sample of the general population. This could introduce bias in the normative values derived from this cohort, potentially affecting the generalizability of the findings to a broader, non-diabetes at-risk population.

## Conclusion

This study reveals a significant portion of the NGT population with elevated 1-h PG, which is mainly attributed to the β-cell dysfunction. The findings suggest that even within the NGT range, individuals with higher 1-h PG may have underlying metabolic disturbances that predispose them to future metabolic disorders. These results advocate for a more nuanced approach in assessing risks associated with glucose levels that are traditionally considered normal.

## Data Availability

The raw data supporting the conclusions of this article will be made available by the authors, without undue reservation.
